# Dynamics of major histocompatibility complex class II-positive cells in the postischemic brain - influence of levodopa treatment

**DOI:** 10.1186/s12974-014-0145-z

**Published:** 2014-08-23

**Authors:** Enida Kuric, Karsten Ruscher

**Affiliations:** Laboratory for Experimental Brain Research, Division of Neurosurgery, Department of Clinical Sciences, Lund University, BMC A13, S-22184 Lund, Sweden

**Keywords:** Cytokine, Inflammation, Levodopa, MHC class II, Microglia, Stroke recovery

## Abstract

**Background:**

Cerebral ischemia activates both the innate and the adaptive immune response, the latter being activated within days after the stroke onset and triggered by the recognition of foreign antigens.

**Methods:**

In this study we have investigated the phenotype of antigen presenting cells and the levels of associated major histocompatibility complex class II (MHC II) molecules in the postischemic brain after transient occlusion of the middle cerebral artery (tMCAO) followed by levodopa/benserazide treatment. Male Sprague Dawley rats were subjected to tMCAO for 105 minutes and received levodopa (20 mg/kg)/benserazide (15 mg/kg) for 5 days starting on day 2 after tMCAO. Thereafter, immune cells were isolated from the ischemic and contralateral hemisphere and analyzed by flow cytometry. Complementarily, the spatiotemporal profile of MHC II-positive (MHC II^+^) cells was studied in the ischemic brain during the first 30 days after tMCAO; protein levels of MHC II and the levels of inflammation associated cytokines were determined in the ischemic hemisphere.

**Results:**

We found that microglia/macrophages represent the main MHC II expressing cell in the postischemic brain one week after tMCAO. No differences in absolute cell numbers were found between levodopa/benserazide and vehicle-treated animals. In contrast, MHC II protein levels were significant downregulated in the ischemic infarct core by levodopa/benserazide treatment. This reduction was accompanied by reduced levels of IFN-γ, TNF-α and IL-4 in the ischemic hemisphere. In the contralateral hemisphere, we exclusively detected MHC II^+^ cells in the corpus callosum. Interestingly, the number of cells was increased by treatment with levodopa/benserazide independent from the infarct size 14 days after tMCAO.

**Conclusions:**

Results suggest that dopamine signaling is involved in the adaptive immune response after stroke and involves microglia/macrophages.

## Background

A long-lasting inflammatory response is observed in the postischemic brain [1] involving the stepwise activation of the innate and the adaptive immune systems [2]. Inflammation initially is triggered by the release of pro-inflammatory molecules such as danger associated molecules (DAMPs), and propagated by pro-inflammatory cytokines and chemokines [3]. Subsequently, accumulation of activated microglial cells in the ischemic territory contribute to encapsulate infarcted tissue and to clear cellular debris [4-6]. Upon microglial activation, immune related molecules such as major histocompatibility complex II (MHC II) are upregulated and involved in antigen presentation and thus in the interaction with peripheral immune cells. MHC II-positive (MHC II^+^) cells are present in the ipsilesional hemisphere during the first month following transient occlusion of the middle cerebral artery [5].

In addition to the ischemic lesion, disruption of neuronal tracts between lesioned and remote brain areas ipsi- and contralateral to the lesioned hemisphere have a significant impact on recovery [7]. These also include interhemispheric connections through the corpus callosum (CC) which contains a large number of myelinated axonal fibers originating from specific cortical areas mainly located in intermediate cortical layers [8]. Disruption of neuronal circuits may go along with delayed degeneration of axonal fibers and possibly with an immune cell response [9]. This idea is supported by studies showing the presence of MHC II^+^ cells in white matter tracts in association with degenerating myelinated axons following injury [5,10]. Moreover, it has been shown that immunoreactivity to myelin basic protein is increased in lymphoid organs of stroke patients which was correlated with worse outcome [11].

Previous studies indicate that dopamine exerts immunomodulatory actions through activation of respective receptors expressed in immune cells [12,13]. In addition, beneficial effects on recovery of lost motor function have been demonstrated in stroke patients treated with levodopa in combination with forced physical training [14,15]. We have demonstrated that delayed treatment with levodopa of rats subjected to transient occlusion of the middle cerebral artery (tMCAO) improves recovery of lost function [16]. Moreover, our studies also suggest that glial and immune cells are involved in levodopa-mediated recovery- enhancing effects [17,18].

The dynamics of immune cell accumulation have been characterized by several independent studies showing a coordinated accumulation of immune cell populations during the first weeks after experimental stroke [19-21]. However, pharmacological modulation by levodopa/benserazide on immune cell infiltration into the postischemic brain after stroke has not been investigated. The present study, therefore, was conducted to investigate if levodopa/benserazide treatment affects poststroke inflammation.

## Materials and methods

### Ethics statement

All animal experiments were carried out with the approval of the Malmö-Lund Ethical Committee and followed the ARRIVE guidelines.

### Transient rat middle cerebral artery occlusion (tMCAO)

tMCAO was induced for 105 minutes as described previously [22]. Male Sprague Dawley rats (325 to 350 g, Harlan Scandinavia, Denmark) were housed under diurnal light conditions and were fasted for 12 hours before surgery. Physiological parameters - arterial blood pressure and gases after tail artery cannulation and insertion of a catheter (SIMS Portex, Hythe, UK) for rectal body temperature - were measured and controlled within their physiological range during surgery. To monitor regional blood flow, an optical fiber probe (Probe 318-I; Perimed, Stockholm, Sweden) was mounted on the skull above the middle cerebral artery (MCA) and connected to a laser Doppler flow meter (Periflux System 5000; Perimed, Stockholm, Sweden). Rats only were included in the study if regional blood flow decreased by at least 70% immediately after the filament was placed; the regional blood flow reduction was sustained during the entire occlusion time (105 minutes); and if the regional blood flow was recovered within 5 minutes after the filament was removed. The same protocol was applied to sham operated rats except that no filament was advanced to occlude the MCA. Experiments were carried out in accordance to the ARRIVE guidelines. For the present investigation, in total 68 Sprague Dawley rats have been operated on. Eighteen rats died during the first 48 hours after tMCAO before randomization; thereafter, none of the animals died until the endpoint of the study. tMCAO for 120 minutes was performed in male Wistar rats (325 to 350 g, HsdBrlHan, Harlan Scandinavia, Denmark, 325 to 350 g, Taconic, Sweden) for immunohistochemistry and immunofluorescence analyses [22].

### Study design and treatment protocol

Sprague Dawley rats were randomized and the studies were performed in a blinded fashion to the investigator who performed the surgeries and behavior assessments. Loss of sensorimotor function was assessed by the rotating pole test and the Garcia test 48 hours after tMCAO [22]. Only animals with a significant loss of function were assigned into the following treatment groups: sham vehicle n = 8, sham levodopa/benserazide n = 7, tMCAO vehicle n = 10 and tMCAO levodopa/benserazide n = 12. Treatment was initiated on day 2 after tMCAO and for an additional 4 days rats received daily 20 mg/kg levodopa combined with 15 mg/kg benserazide (Sigma-Aldrich, Deisenhofen, Germany) or saline (vehicle) by intraperitoneal injection. On day 7 after stroke onset, rats were sacrificed and brains have been processed for fluorescence-activated cell sorting (FACS) analysis. The study design was chosen so that treatment would not interfere with acute cell death mechanisms which essentially have finished at 48 hours after stroke onset. The rationale to test a dosage of 20 mg/kg of levodopa is based on our previous study showing improved recovery after tMCAO [16].

Wistar rats were randomized and treated as described previously [16], sacrificed 14 days after the surgery. Tissue from the same set of those animals was used in the present study. In particular, for western blots, immunohistochemistry, immunoassay and quantification of the number of MHC II^+^ cells, the following number of animals have been included: sham vehicle n = 4, sham LD n = 4, tMCAO vehicle n = 8, tMCAO LD n = 8. To study the spatiotemporal profile of MHC II^+^ expressing cells in the postischemic brain, coronal sections from Wistar rats subjected to tMCAO for 120 minutes were used as described previously [22]. At least sections from 3 animals per time point (2 days, 4 days, 7 days, 14 days and 30 days) were analyzed.

### Isolation of immune cells from brain tissue

Immune cells were prepared as described previously [23]. In brief, rats have been anesthetized with isoflurane and blood cells were removed from cerebral vessels by perfusion with saline for four minutes. Thereafter, brains were obtained, hemispheres separated and meninges were removed from the brains. Hemispheres were dissociated in ice-cold Hank’s balanced salt solution (HBSS, supplemented with 0.2% BSA (Sigma-Aldrich, Deisenhofen, Germany), 0.01% ethylenediaminetetraacetic acid (EDTA, Sigma-Aldrich, Deisenhofen, Germany) using a Dounce homogenizer and passed through a 40-μm nylon cell strainer (BD Biosciences, Stockholm, Sweden). After centrifugation at 400 × g at room temperature (rt) for 10 minutes, the pellet was resuspended in a Percoll solution (30% in HBSS) and overlaid on a gradient of a 37% and 70% Percoll solution. The subsequent centrifugation was carried out at 500 × g at rt for 20 minutes. Immune cells were carefully collected at the 37% to 70% interface and washed with HBSS containing 10% FBS. After centrifugation at 400 × g at rt for 10 minutes), cells were resuspended in 500 μL PBS containing 0.2% BSA.

### Fluorescence activated cell sorter analysis

One hundred microliters of sample obtained from brain were used per staining panel and stained with mouse anti rat MHC II phycoerythrin (PE) conjugated, anti rat cluster of differentiation (CD) 11b fluorescein isothiocyanate (FITC) conjugated, anti rat CD11c Alexa Fluor 647 conjugated, anti rat CD45-Alexa eFluoro 700 or anti rat MHC II PE conjugated, anti rat CD80 FITC conjugated, and anti rat CD86 phycoerythrin cyanine 7 (PE-Cy7) and anti rat CD11c Alexa Fluor 647 conjugated (diluted 1:200, BD Biosciences, San Jose, CA, USA). After washing and centrifugation at 1,006 × g for 1 minute, cells were fixed with (Cytofix/Cytoperm™, BD Biosciences, San Jose, CA, USA) following the manufacturers protocol. Unstained cells and fluorescence minus one (FMO) controls were used in parallel.

**Figure 1:**
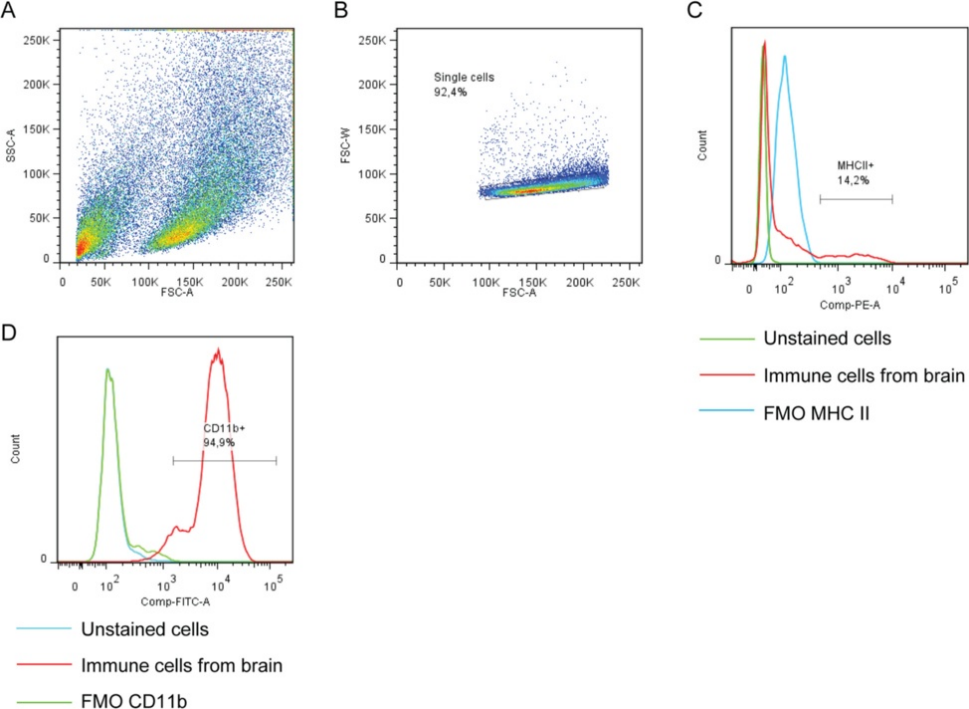
**Experimental design.** Representative scatter plots and gating strategies for immune cell analysis in brains from rats subjected to tMCAO. **(A)** Cells were first gated for lymphocytes (SSC-A versus FSC-A) and **(B)** singlets (FSC-W versus FSC-A). Thereafter, surface expression of **(C)** MHC class II or **(D)** CD11b was determined from this gated population. Abbreviations: forward side scatter (FSC); side scatter (SSC); fluorescence minus one (FMO).

Forty-eight hours after fixation, cells were analyzed and data collected using FACSAria III and FACS Diva software (BD Biosciences, San Jose, CA, USA). Morphological analysis was performed by using the forward side scatter (FSC) and side scatter (SSC) parameters. Up to 200,000 events were acquired and FSC threshold was set to 20,000. Data analysis was performed by using the FlowJo (7.6.5 version, Tree Star, Ashland, OR, USA) software. Cells were morphologically identified by FSC and SSC and doublets were discriminated and further divided by antigen positivity in respect to unstained cells and FMO controls (Figure 1A and B). Microglia/macrophages and dendritic cells were identified being CD45^+^ and further analyzed for CD11b and CD11c expression. Microglia/macrophages were defined being (CD45^+^CD11b^+^CD11c^−^) and dendritic cells being (CD45^+^CD11b^+^CD11c^+^) according to Gelderblom *et al*., [19].

### Preparation of protein extracts from brain tissue

Whole cellular protein extracts were prepared as described previously [24]. In brief, tissue from the infarct core was homogenized by a Dounce homogenizer in lysis buffer containing 20 mM Tris (pH 7.5), 150 mM NaCl, 1 mM EDTA, 1 mM ethylene glycol tetraacetic acid (EGTA), 1 mM phenylmethanesulfonyl fluoride (PMSF), 2.5 mM sodium pyrophosphate, 1 mM β-glycerolphosphate, 1 mM sodium orthovanadate supplemented with protease inhibitor cocktail (catalog # P2714, Sigma-Aldrich, Deisenhofen, Germany). After 15 minutes of incubation on ice, the homogenates were centrifuged (18,000 x g at 4°C for 20 minutes) and the supernatant was collected for further analysis. Total protein concentrations were determined with the Bradford assay using BSA as standard.

### Cytokine analysis from brain extracts

Brain extracts were obtained from the peri-infarct area and the infarct core of rats subjected either to tMCAO or sham operation, respectively, and treated either with levodopa/benserazide (20 mg/kg) or vehicle (saline) for 12 days in total. Samples were diluted with lysis buffer and analyzed for the following cytokines: IL-1β, chemokine (C-X-C motif) ligand 1 (CXCL1), IL-4, IL-5, TNF-α, IFN-γ and IL-13 by a multiplex immunoassay kit according to manufacturer’s protocol (Mesoscale, Gaithersburg, MD, USA) and as described previously [22].

### Western blotting

Twenty micrograms of protein were separated on a 10% acrylamide gel. Thereafter, proteins were transferred onto polyvinylidene difluoride (PVDF) membranes and blocked with a solution containing 5% non-fat dry milk, 2% normal horse serum and 2% BSA. Following blocking, the membranes were incubated with mouse anti rat MHC II (Ox-6) (dilution 1:1,000, AbD Serotec GmbH, Düsseldorf, Germany) at 4°C over night. Subsequently, the membranes were incubated with a secondary antibody anti mouse horseradish peroxidase (HRP) conjugated (1:8,000, Sigma-Aldrich, Deisenhofen, Germany) for one hour at rt. The signals were visualized by using a chemiluminescence kit (Merck Millipore, Darmstadt, Germany) and CCD camera (Fujifilm LAS 1000, Fujifilm, Tokyo, Japan). Membranes were stripped and reprobed with anti β-actin HRP conjugated (dilution 1:50,000, Sigma-Aldrich, Deisenhofen, Germany).

### Immunohistochemistry

Paraformaldehyde-fixed free-floating brain sections (thickness 30 μm) were used. After quenching endogenous peroxidase activity and blocking in normal rabbit or donkey serum, the sections were incubated with a mouse anti rat MHC II (diluted to 1:100, AbD Serotec) at 4°C over night. Following rinsing, the sections were incubated with a biotinylated donkey anti mouse antibody (1:500, Jackson ImmunoResearch, Newmarket, UK) for 90 minutes and subsequently with avidin biotin complex (Vector Laboratories, Burlingame, CA, USA). The reaction was carried out by the addition of 3,3-diaminobenzidine (DAB) (Saveen and Werner, Limhamn, Sweden) supplemented with 8% nickel chloride.

### Immunofluorescence

Free-floating, paraformaldehyde-fixed brain sections, were stained with rabbit anti rat dopamine 1 receptor (diluted to 1:200, Abcam, Cambridge, UK) and mouse anti rat MHC II (diluted to 1:100, AbD Serotec, Düsseldorf, Germany) at 4°C overnight after blocking in 5% normal serum for one hour at rt. Thereafter, sections were incubated with secondary swine anti rabbit biotinylated antibody (diluted to 1:400, Jackson ImmunoResearch, Newmarket, UK) for one hour at rt, followed by incubation with Streptavidin Alexa 488 (diluted to 1:400) and donkey anti mouse Cy3 conjugated (diluted to 1:400, Jackson ImmunoResearch, Newmarket, UK) for 90 minutes at rt.

### Quantification of MHC II^+^ cells

Two coronal sections (−0.90 and −1.90 related to bregma) per brain were stained for MHC II as described above. Composite micrographs of the whole contralateral hemisphere were taken by bright field microscopy (Olympus BX61 equipped with a 10-fold objective, Olympus Sverige AB, Solna, Sweden). The CC of the contralateral hemisphere was defined by a medial vertical line. MHC II^+^ cells were counted in the entire CC.

### Statistics

Flow cytometry and cytokine data are displayed as mean ± standard error of the means and tested with analysis of variance (ANOVA) with *post hoc* Bonferroni correction or Fisher’s least significant difference (LSD) test unless otherwise specified using SPSS™ version 21 (IBM, Stockholm, Sweden). Differences in the number of MHC II^+^ cells and levels of MHC II were tested with Student’s *t*-test. In all experiments *P* < 0.05 was considered significant.

## Results

### Dynamics of MHC II^+^ cells in the postischemic brain after levodopa/benserazide treatment

**Figure 2:**
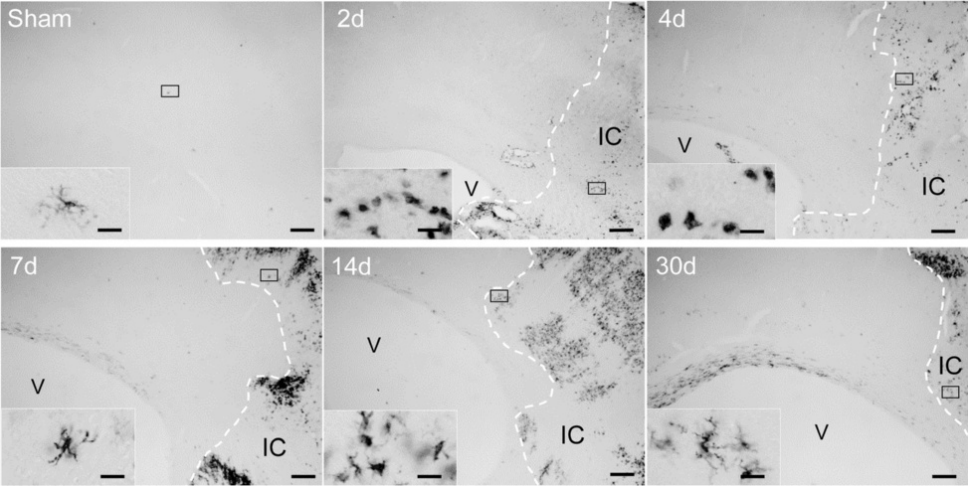
**Spatiotemporal expression of MHC II protein in the ischemic hemisphere.** Spatiotemporal distribution of MHC II expressing cells in the ipsilateral cortex of a sham operated animal and at the indicated time points after tMCAO. The border of the infarct core is delineated by a dotted line. Scale bars: low magnification - 200 μm; higher magnification - 20 μm. Abbreviations: V, lateral ventricle; IC, infarct core.

To evaluate the spatiotemporal expression of immune cell accumulation rats were subjected to tMCAO for 105 minutes and at different time points coronal sections were stained for MHC II, a protein expressed on different immune cell populations [25]. As shown in Figure 2, we observed very few scattered and ramified cells in brains of sham operated rats. In contrast, an accumulation of ameboid cells was evident in the ischemic territory during the first four days after tMCAO. At later time points (7 days, 14 days and 30 days), MHC II^+^ cells in the ischemic territory consistently displayed a ramified morphology (Figure 2).

**Figure 3:**
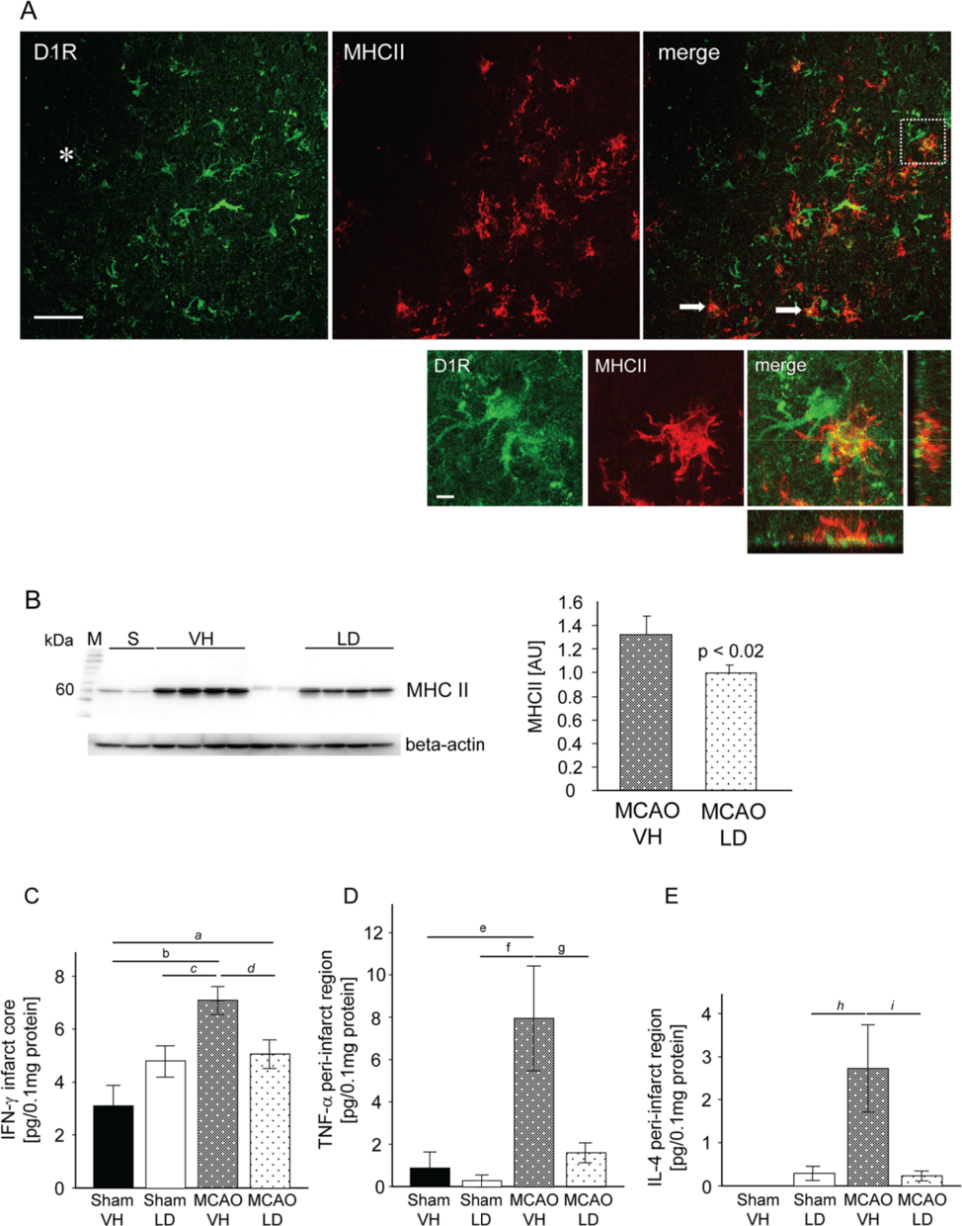
**Reduction of MHC II protein and pro-inflammatory cytokines 14 days after tMCAO. (A)** Co-localization of MHC II (Cy3, red) and D1 receptors (Alexa 488, green) in the peri-infarct area 14 days after tMCAO; two cells are indicated with an arrow. Higher magnification of the area delineated in the merged low power micrograph. The asterisk indicates the infarct core. Scale bars: low magnification - 50 μm; high magnification - 5 μm. **(B)** Western blot analysis for MHC II in the infarct core of rats treated either with vehicle (VH, n = 4) or levodopa/benserazide (LD, n = 4). Levels of MHC II protein are presented as arbitrary units (AU) to the right. Data are displayed as means ± SEM, Student’s *t*-test, *P* < 0.05. **(C)** Levels of IFN-γ in the infarct core, **(D)** TNF-α and **(E)** IL-4 levels in the peri-infarct region from sham operated animals (VH n = 4; LD n = 4) and rats subjected to tMCAO (VH n = 8; LD n = 8). Results are presented as means ± SEM. Statistical differences were tested with two-way ANOVA followed by Bonferroni *post hoc* correction or Fisher’s Least Significant Difference test (italic). Letters denote *P* values; *a*) *P* = *0.043*, b) *P* = 0.002, *c*) *P* = *0.021. d*) *P* = *0.013*, e) *P* = 0.016, f) *P* = 0.009, g) *P* = 0.032, *h*) *P* = *0.030*, *i*) *P* = *0.008*. Abbreviations: M, marker lane (kDa); S, sham operated animals; VH, vehicle; LD, levodopa/benserazide.

Among other cell types [15,16], dopamine 1 (D1) receptors are expressed on a number of MHC II^+^ cells in the ischemic territory (Figure 3A) strongly suggesting that MHC II cells are regulated by treatment with levodopa. Indeed, analysis of MHC II protein from the ischemic core region showed a significant reduction in rats treated with levodopa (5 mg/kg)/benserazide (15 mg/kg) on day 14 after tMCAO (tMCAO VH 1.35 ± 0.17, tMCAO LD 0.98 ± 0.08; arbitrary units) (Figure 3B). This was accompanied by a significant reduction of pro-inflammatory cytokines IFN-γ, TNF-α and IL-4 in the ischemic hemisphere known to induce MHC II expression [26,27] (Figure 3D to F).

**Figure 4:**
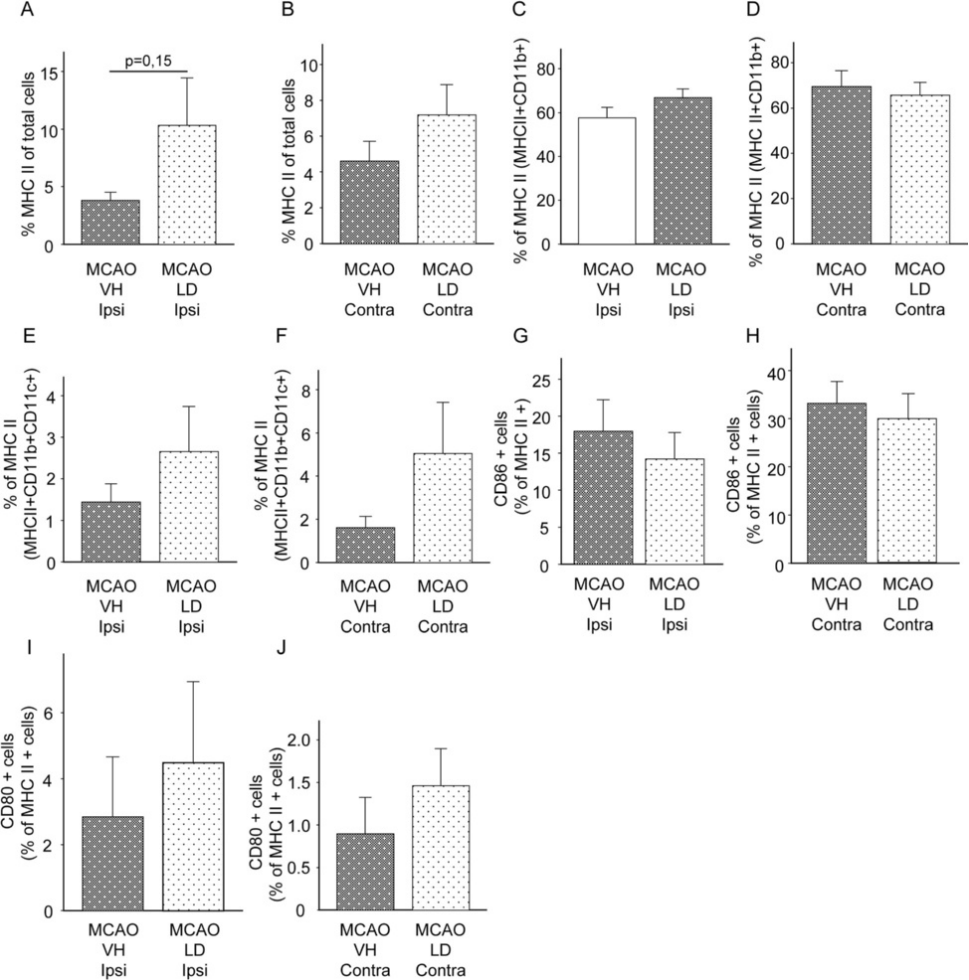
**Phenotyping of MHC II** ^**+**^ **seven days after tMCAO.** Number of MHC II^+^ cells in total in the ipsilateral **(A)** and contralateral **(B)** hemisphere. Data are presented as means ± SEM and a ratio between the number of MHC II^+^ cells to the total number of cells. Number of MHC II^+^ cells co-expressing (CD11b^+^CD45^+^), defined as microglia/macrophages in the ipsilateral **(C)** and contralateral **(D)** hemisphere presented as means ± SEM and as a ratio of CD11b^+^CD45^+^ cells to the total number of MHC II^+^ cells. Number of MHC II^+^ cells co-expressing CD11b^+^CD11c^+^CD45^+^, defined as myeloid dendritic cells in the ipsilateral **(E)** and contralateral **(F)** hemisphere presented as means ± SEM and as a ratio of CD11b^+^CD11c^+^CD45^+^ cells to the total number of MHC II^+^ cells. Co-expression of stimulatory molecules, CD86 in the ipsilateral **(G)** and contralateral hemisphere **(H)** and CD80 in the ipsilateral **(I)** and contralateral hemisphere **(J)** presented as means ± SEM and as ratio to the total number of MHC II^+^ cells. Abbreviations: VH, vehicle; LD, levodopa/benserazide; Ipsi, ipsilateral hemisphere; Contra, contralateral hemisphere.

Delayed accumulation of immune cells and expression of MHC II protein in the postischemic brain prompted us to perform flow cytometry analysis of immune cells in brain tissues of rats one week after tMCAO. Figures 4A and B demonstrate that treatment with levodopa/benserazide had no effect on the number of MHC II^+^ cells in the ischemic but also the contralateral hemisphere (absolute numbers of cells are shown in Table 1). The majority of MHC II^+^ cells in the ischemic and contralateral hemisphere were identified as microglia/macrophages by the expression of CD45 and CD11b, (CD11b^+^CD45^+^MHC II^+^) (Figure 4C and D, Table 1). Treatment with levodopa/benserazide had no effect on the number of microglia/macrophages. In addition, a MHC II expressing cell population was defined by the antigens MHCII, CD11b, CD11c and CD45, respectively, and was identified as myeloid dendritic cells in both ipsilateral and contralateral hemisphere (Figure 4E and F). A significant portion of MHC II^+^ cells co-expressed the stimulatory molecules CD86 (Figure 4G and H) and CD80 (Figure 4I and J) necessary for the effector cell response upon interaction between immune cells, suggesting that these cells represent professional antigen presenting cells (APCs).

**Table 1:** **Number of MHC II** ^**+**^ **cells in the brain after tMCAO**

**MCAO Ipsilateral**	**MHC II** ^**+**^	**MHC II** ^**+**^ **CD11b** ^**+**^	**MHC II** ^**+**^ **CD11b** ^**+**^ **CD11c** ^**+**^
MCAO vh	2,732 ± 737	1,692 ± 540	36 ± 13
MCAO LD	3,693 ± 1,235	2,680 ± 1,060	77 ± 27
MCAO Contralateral	MHC II^+^	MHC II^+^CD11b^+^	MHC II^+^CD11b^+^CD11c^+^
MCAO vh	880 ± 215	650 ± 192	14 ± 4
MCAO LD	1,458 ± 462	1,048 ± 368	61 ± 25

Number of MHC II^+^ cells in brains from rats subjected to tMCAO for 105 minutes and treated either with saline (MCAO vh) or levodopa/benserazide (MCAO LD). Results are shown as absolute numbers ± SEM.

**Figure 5:**
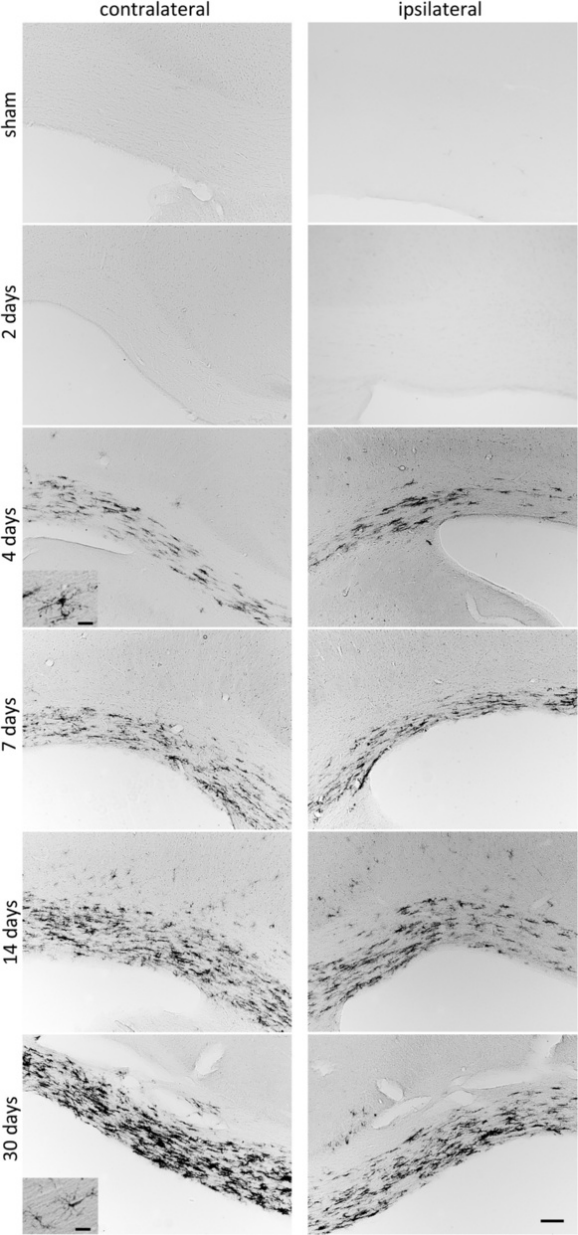
**MHC II** ^**+**^ **cells in the corpus callosum (CC) after tMCAO.** Temporal expression of MHC II in the CC contralateral and ipsilateral to the lesioned hemisphere at different time points after tMCAO and a sham operated animal, respectively. Scale bars: lower magnification - 100 μM; higher magnification - 20 μM.

**Figure 6:**
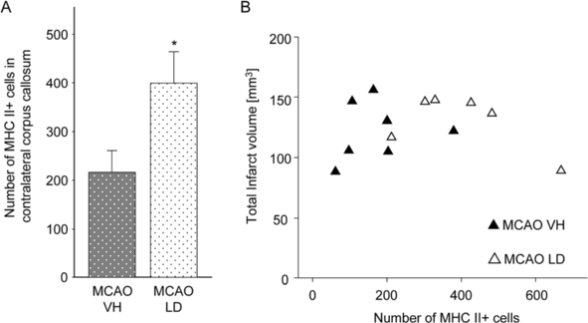
**Effect of levodopa/benserazide treatment on MHC II** ^**+**^ **cells in the corpus callosum (CC) after tMCAO. (A)** Number of MHC II^+^ cells in the CC contralateral to the lesioned hemisphere of rats treated with vehicle (n = 7) or levodopa/benserazide (n = 6). Data are presented as means ± SEM and statistical difference was tested with the Student’s *t*-test, *P* < 0.05. **(B)** Correlation between infarct volume presented in mm^3^ and the absolute number of MHC II^+^ cells. Abbreviations: VH, vehicle; LD, levodopa/benserazide.

Interestingly, further analysis of brain sections revealed the selective appearance of MHC II^+^ cells in the CC contralateral to the ischemic hemisphere (Figure 5). Cell accumulation was detectable as early as 4 days after stroke onset and onwards and cells were present until the study endpoint at 30 days after tMCAO. No differences between animal strains were observed on day 14 after stroke (data not shown). Cells displayed a ramified morphology and were mostly present in the CC. However, scattered positive cells were also found in the striatum of the contralateral hemisphere. Quantification revealed a higher number of MHC II^+^ cells in rats treated with levodopa/benserazide (399 ± 66 cells, saline treatment 216 ± 45 cells, Figure 6A). Importantly, the number of MHC II^+^ cells in the contralateral CC did not correlate with the infarct volume (Figure 6B) and thus not the size of injury. These results illustrate that the delayed cellular inflammatory response involving MHC II^+^ cells includes the contralateral callosum, a brain region rich in axonal fibers and connecting both hemispheres, after stroke.

## Discussion

In the present study we investigated if treatment with levodopa/benserazide affects delayed inflammation after stroke, which is the number of MHC II^+^ cells and levels of pro-inflammatory molecules in the postischemic brain. We found that MHC II^+^ cells accumulate in the ischemic hemisphere independent from the treatment while levodopa/benserazide treatment significantly downregulated MHC II proteins in the ischemic territory. Moreover, we detected a delayed accumulation of MHC II^+^ cells in the CC ipsi- and contralateral to the lesioned hemisphere and found that the number of these cells in the contralateral CC is increased by the treatment.

### Dynamics of immune cell accumulation in the postischemic brain - influence of levodopa treatment

Our studies show that treatment with levodopa/benserazide is effective in two rat strains subjected to tMCAO. Moreover, postischemic immune cell accumulation to the brain observed in vehicle-treated Sprague Dawley rats subjected to tMCAO are similar to those found in Wistar rats subjected to tMCAO [23]; hence, the time of occlusion necessary to obtain similar lesions differed between the strains. Both strains have been compared in experimental stroke models [28,29]. A higher variability of lesions was observed in Wistar rats subjected to photothrombosis and in Sprague Dawley rats subjected to tMCAO with a higher survival rate after the insult. We have extensively tested times of MCA occlusion in both rat strains and similar lesions and functional deficits were obtained using 120 minutes in Wistar rats and 105 minutes in Sprague Dawley rats. While Wistar rats showed a high variability in lesion size, consistent infarctions and higher survival rates were observed in Sprague Dawley rats. Consistent and large lesions are essential to obtain a sufficient inflammatory response including the accumulation of high enough immune cell counts from individual animals.

Based on immunohistochemistry and FACS analysis we could confirm a delayed accumulation of MHC II^+^ cells in the postischemic brain after tMCAO [21] and show that the majority of MHC II^+^ cells are microglia/macrophages identified by co-expression of CD11b and CD45 [5]. Only a minority of the MHC II^+^ cell population was identified as dendritic cells. A portion of MHC II^+^ cells co-expressed CD80 or CD86, molecules necessary for signal transduction between immune cells and identifying these cells as professional antigen presenting cells [30]. Our findings are in contrast to a recent study demonstrating a widespread invasion of dendritic cells expressing the co-stimulatory molecule CD80 after tMCAO. Differences might be due to species differences and the use of a CD11c driven enhanced yellow fluorescent protein (EYFP) as an overall dendritic cell marker [31].

Additional immunofluorescence analyses showed that a number of MHC II^+^ cells express D1 receptors emphasizing their susceptibility to levodopa treatment. Together with the lack of D2 and D3 receptor expression (data not shown) this finding supports mechanisms dependent on the activation of D1 receptors in these cells. Our experiments showed different effects of levodopa/benserazide treatment in regard to the levels of MHC II proteins and the number of MHC II^+^ cells in the lesioned hemisphere. While MHC II proteins were significantly downregulated we found no differences in the number of MHC II^+^ cells between vehicle and levodopa/benserazide-treated animals. The expression of MHC II molecules depends on presentation of antigens to immune cells such as microglia and involves the activation of intracellular signalling cascades in respective cells [32]. On the other hand, even with a very low number of MHC II molecules presented on the surface, cells were determined as MHC II^+^. The findings are not contradictory because also low numbers of MHC II molecules found in levodopa/benserazide-treated rats are presented on the cell surface and, therefore, these cells were counted as MHC II^+^. Hence, this assumption is only valid if no differences in posttranslational intracellular transport, integration of MHC II molecules on the cell surface and the same overall accumulation of immune cells in the lesioned hemisphere exist as shown in Figure 4. The two approaches to assess MHC II, therefore, can only be considered as complementary.

The regulation of MHC II expression in microglial cells has not been investigated in detail. It has been shown that infusion of IFN-γ increases the expression of MHC II in microglia [33]. Thus, long-term suppression of IFN-γ in the ischemic hemisphere by levodopa/benserazide treatment may downregulate MHC II proteins. A possible mechanism to reduce the expression of MHC II molecules by dopamine signaling might be via inhibitory actions of the protein kinase A (PKA). As previously shown, activation of D1 receptors leads to phosphorylation of PKA [34] which subsequently inhibits CIITA, a positive regulator of MHC II gene transcription [35].

Pro-inflammatory cytokines are also expressed in other resident brain cells such as neurons or astrocytes [36,37]. Treatment with levodopa/benserazide also has effects on the function of these cells expressing dopamine receptors including a reduction of cytokine release. Paracrine communication of cells in turn may affect surrounding immune cells such as MHC II^+^ cells. Taken together, our results point towards immunomodulatory effects involving the expression of MHC II molecules by levodopa treatment in the postischemic brain, possibly by a reduction of pro-inflammatory cytokines.

### Putative functions of MHC II^+^ cells in the corpus callosum

The CC represents one of the principal connections in the communication between the two hemispheres [8]. Here, for the first time we describe the delayed accumulation of MHC II^+^ cells in the CC of the ipsi- and contralateral hemisphere after tMCAO. Further characterization by FACS analysis revealed that the majority of these cells are CD11b^+^ microglia. In addition, we identified a small portion of cells co-expressing CD11b and CD11c indicative for myeloid dendritic cells [19]. A possible functional relevance of these cells might be that due to ischemic injury, anterograde as well as retrograde degeneration of neuronal fibers takes place, and accumulation of MHC II^+^ cells might either perpetuate neurodegeneration or provide a protective/trophic support to neurons [38]. Quantification of MHC II^+^ cells in the contralateral CC showed an increased number of cells in animals treated with levodopa/benserazide independent from the infarct volume which was not affected by the treatment [16]. Since MHC II^+^ cells appear ramified and activated [39] and do not display an ameboid phagocyte-like morphology, as observed in the infarct core, a neurotrophic function and support in remyelination might be possible [36]. Hence, we cannot exclude phagocytosis of cellular debris.

In conclusion, our results demonstrate that dopamine attenuates the inflammatory response in the ischemic hemisphere. In addition, our data show an accumulation of MHC II^+^ cells in the CC of the hemisphere contralateral and ipsilateral to the ischemic lesion. Interestingly, the number of cells was significantly elevated by treatment with levodopa/benserazide suggesting that these cells exert beneficial actions contributing to recovery after stroke. Together, both components of dopamine actions might be exploited in future recovery-enhancing stroke therapies.

## Abbreviations

CC: corpus callosum
MHC II: major histocompatibility complex class II
tMCAO: transient Middle cerebral artery occlusion
FACS: fluorescence-activated cell sorting
HBSS: Hank’s balanced salt solution
EDTA: ethylenediaminetetraacetic acid
CD: cluster of differentiation
LD: levodopa
BSA: bovine serum albumin
FBS: fetal bovine serum
PBS: phosphate buffered saline
rt: room temperature
PE: phycoerythrin
CXCL1: chemokine (C-X-C motif) ligand 1
FITC: Fluorescein isothiocyanate
IL: interleukin
IFN: interferon
FMO: fluorescence minus one
FSC: forward side scatter
SSC: side scatter
EGTA: ethylene glycol tetraacetic acid
PMSF: phenylmethanesulfonyl fluoride
PVDF: polyvinylidene difluoride
TNF: tumor necrosis factor
HRP: horseradish peroxidase
CCD: charged-coupled device
DAB: 3,3-diaminobenzidine
ANOVA: analysis of variance
LSD: least significant difference
Cy: cyanine, VH, vehicle
AU: arbitrary unit
SEM: standard error of the mean
D: dopamine

## References

[1] Lalancette-Hebert M, Phaneuf D, Soucy G, Weng YC, Kriz J (2009). Live imaging of Toll-like receptor 2 response in cerebral ischaemia reveals a role of olfactory bulb microglia as modulators of inflammation. Brain.

[2] Kamel H, Iadecola C (2012). Brain-immune interactions and ischemic stroke: clinical implications. Arch Neurol.

[3] Shichita T, Hasegawa E, Kimura A, Morita R, Sakaguchi R, Takada I, Sekiya T, Ooboshi H, Kitazono T, Yanagawa T, Ishii T, Takahashi H, Mori S, Nishibori M, Kuroda K, Akira S, Miyake K, Yoshimura A (2012). Peroxiredoxin family proteins are key initiators of post-ischemic inflammation in the brain. Nat Med.

[4] Kreutzberg GW (1996). Microglia: a sensor for pathological events in the CNS. Trends Neurosci.

[5] Morioka T, Kalehua AN, Streit WJ (1993). Characterization of microglial reaction after middle cerebral artery occlusion in rat brain. J Comp Neurol.

[6] Smirkin A, Matsumoto H, Takahashi H, Inoue A, Tagawa M, Ohue S, Watanabe H, Yano H, Kumon Y, Ohnishi T, Tanaka J (2010). Iba1 (+)/NG2 (+) macrophage-like cells expressing a variety of neuroprotective factors ameliorate ischemic damage of the brain. J Cereb Blood Flow Metab.

[7] Wang LE, Tittgemeyer M, Imperati D, Diekhoff S, Ameli M, Fink GR, Grefkes C (2012). Degeneration of corpus callosum and recovery of motor function after stroke: a multimodal magnetic resonance imaging study. Hum Brain Mapp.

[8] Fame RM, MacDonald JL, Macklis JD (2011). Development, specification, and diversity of callosal projection neurons. Trends Neurosci.

[9] Zhang J, Zhang Y, Xing S, Liang Z, Zeng J (2012). Secondary neurodegeneration in remote regions after focal cerebral infarction: a new target for stroke management?. Stroke.

[10] Smetanka AM, Yee KT, Lund RD (1990). Differential induction of class I and II MHC antigen expression by degenerating myelinated and unmyelinated axons. Brain Res.

[11] Planas AM, Gomez-Choco M, Urra X, Gorina R, Caballero M, Chamorro A (2012). Brain-derived antigens in lymphoid tissue of patients with acute stroke. J Immunol.

[12] Basu S, Dasgupta PS (2000). Dopamine, a neurotransmitter, influences the immune system. J Neuroimmunol.

[13] Sarkar C, Basu B, Chakroborty D, Dasgupta PS, Basu S (2010). The immunoregulatory role of dopamine: an update. Brain Behav Immun.

[14] Scheidtmann K, Fries W, Muller F, Koenig E (2001). Effect of levodopa in combination with physiotherapy on functional motor recovery after stroke: a prospective, randomised, double-blind study. Lancet.

[15] Acler M, Fiaschi A, Manganotti P (2009). Long-term levodopa administration in chronic stroke patients. A clinical and neurophysiologic single-blind placebo-controlled cross-over pilot study. Restor Neurol Neurosci.

[16] Ruscher K, Kuric E, Wieloch T (2012). Levodopa treatment improves functional recovery after experimental stroke. Stroke.

[17] Kuric E, Wieloch T, Ruscher K (2013). Dopamine receptor activation increases glial cell line-derived neurotrophic factor in experimental stroke. Exp Neurol.

[18] Kuric E, Ruscher K (2014). Reversal of stroke induced lymphocytopenia by levodopa/benserazide treatment. J Neuroimmunol.

[19] Gelderblom M, Leypoldt F, Steinbach K, Behrens D, Choe CU, Siler DA, Arumugam TV, Orthey E, Gerloff C, Tolosa E, Magnus T (2009). Temporal and spatial dynamics of cerebral immune cell accumulation in stroke. Stroke.

[20] Campanella M, Sciorati C, Tarozzo G, Beltramo M (2002). Flow cytometric analysis of inflammatory cells in ischemic rat brain. Stroke.

[21] Liesz A, Zhou W, Mracsko E, Karcher S, Bauer H, Schwarting S, Sun L, Bruder D, Stegemann S, Cerwenka A, Sommer C, Dalpke AH, Veltkamp R (2011). Inhibition of lymphocyte trafficking shields the brain against deleterious neuroinflammation after stroke. Brain.

[22] Ruscher K, Johannesson E, Brugiere E, Erickson A, Rickhag M, Wieloch T (2009). Enriched environment reduces apolipoprotein E (ApoE) in reactive astrocytes and attenuates inflammation of the peri-infarct tissue after experimental stroke. J Cereb Blood Flow Metab.

[23] Ruscher K, Kuric E, Liu Y, Walter HL, Issazadeh-Navikas S, Englund E, Wieloch T (2013). Inhibition of CXCL12 signaling attenuates the postischemic immune response and improves functional recovery after stroke. J Cereb Blood Flow Metab.

[24] Ruscher K, Freyer D, Karsch M, Isaev N, Megow D, Sawitzki B, Priller J, Dirnagl U, Meisel A: **Erythropoietin is a paracrine mediator of ischemic tolerance in the brain: evidence from an*****in vitro*****model.** *J Neurosci* 2002, **22:**10291–10301. 10.1523/JNEUROSCI.22-23-10291.2002 PMC6758760 12451129

[25] Perry VH (1998). A revised view of the central nervous system microenvironment and major histocompatibility complex class II antigen presentation. J Neuroimmunol.

[26] Peng ZC, Kristensson K, Bentivoglio M (1998). Distribution and temporal regulation of the immune response in the rat brain to intracerebroventricular injection of interferon-gamma. Exp Neurol.

[27] Panek RB, Benveniste EN (1995). Class II MHC gene expression in microglia. Regulation by the cytokines IFN-gamma, TNF-alpha, and TGF-beta. J Immunol.

[28] Aspey BS, Taylor FL, Terruli M, Harrison MJ (2000). Temporary middle cerebral artery occlusion in the rat: consistent protocol for a model of stroke and reperfusion. Neuropathol Appl Neurobiol.

[29] Markgraf CG, Kraydieh S, Prado R, Watson BD, Dietrich WD, Ginsberg MD (1993). Comparative histopathologic consequences of photothrombotic occlusion of the distal middle cerebral artery in Sprague–Dawley and Wistar rats. Stroke.

[30] O’Keefe GM, Nguyen VT, Benveniste EN (2002). Regulation and function of class II major histocompatibility complex, CD40, and B7 expression in macrophages and microglia: implications in neurological diseases. J Neurovirol.

[31] Felger JC, Abe T, Kaunzner UW, Gottfried-Blackmore A, Gal-Toth J, McEwen BS, Iadecola C, Bulloch K (2010). Brain dendritic cells in ischemic stroke: time course, activation state, and origin. Brain Behav Immun.

[32] Hanisch UK (2013). Proteins in microglial activation-inputs and outputs by subsets. Curr Protein Pept Sci.

[33] Vass K, Lassmann H (1990). Intrathecal application of interferon gamma. Progressive appearance of MHC antigens within the rat nervous system. Am J Pathol.

[34] Svenningsson P, Nishi A, Fisone G, Girault JA, Nairn AC, Greengard P (2004). DARPP-32: an integrator of neurotransmission. Annu Rev Pharmacol Toxicol.

[35] Li G, Harton JA, Zhu X, Ting JP (2001). Downregulation of CIITA function by protein kinase a (PKA)-mediated phosphorylation: mechanism of prostaglandin E, cyclic AMP, and PKA inhibition of class II major histocompatibility complex expression in monocytic lines. Mol Cell Biol.

[36] Ohtaki H, Yin L, Nakamachi T, Dohi K, Kudo Y, Makino R, Shioda S (2004). Expression of tumor necrosis factor alpha in nerve fibers and oligodendrocytes after transient focal ischemia in mice. Neurosci Lett.

[37] Denes A, Ferenczi S, Halasz J, Kornyei Z, Kovacs KJ (2008). Role of CX3CR1 (fractalkine receptor) in brain damage and inflammation induced by focal cerebral ischemia in mouse. J Cereb Blood Flow Metab.

[38] Arnett HA, Wang Y, Matsushima GK, Suzuki K, Ting JP (2003). Functional genomic analysis of remyelination reveals importance of inflammation in oligodendrocyte regeneration. J Neurosci.

[39] Ito D, Tanaka K, Suzuki S, Dembo T, Fukuuchi Y (2001). Enhanced expression of Iba1, ionized calcium-binding adapter molecule 1, after transient focal cerebral ischemia in rat brain. Stroke.

